# Effectiveness of aspirin compare with heparin plus aspirin in recurrent pregnancy loss treatment: A Quasi experimental study

**Published:** 2014-01

**Affiliations:** 1 *Department of Medical Genetics, Research and Clinical Center for Infertility, Shahid Sadoughi University of Medical Sciences, Yazd, Iran.*; 2 *Department of Obstetrics and Gynecology, Research and Clinical Center for Infertility, Shahid Sadoughi University of Medical Sciences, Yazd, Iran.*

**Keywords:** *Thrombophilia*, *Recurrent miscarriage*, *Anticoagulant*, *Live birth*, *Aspirin*, *Heparin*

## Abstract

**Background:** Using aspirin, heparin, or both in women with unexplained recurrent miscarriage could be useful, because this problem might be initiated by thrombosis in decidual vessels.

**Objective:** To investigate the association between thrombophilia and unexplained recurrent miscarriage and to evaluate the efficacy of anticoagulant treatment.

**Materials and Methods:** In this quasi experimental, we enrolled 520 women, who had a history of recurrent miscarriage. Two hundred fifty two women with unexplained recurrent miscarriage were assigned to receive aspirin (80 mg daily) for two month before pregnancy and after confirmation of a viable pregnancy until 36 weeks of gestation or receive aspirin, as the same, plus heparin (5000 unit twice a day) subcutaneously after confirmation of viable pregnancy until 4 weeks after delivery. Type of medication was chosen for each woman according to number of abortion and age.

**Results:** Live-birth rates did not different significantly among the two study groups. The proportions of women who gave birth to a live normal infant were 74.5% in the group receiving aspirin plus heparin (combination-therapy group) and 79.8% in the aspirin group.

**Conclusion:** Live-birth rates did not different significantly among the two study groups. So, using aspirin or aspirin plus heparin did not change pregnancy rate in these patients. Using aspirin is easier than injecting heparin which should be chosen case by case.

This article extracted from M.Sc. thesis. (Tahereh Jahaninejad)

Registration ID in IRCT: IRCT2013102315123N1

## Introduction

Recurrent pregnancy loss (RPL) is defined as two or more consecutive pregnancy losses before twenty week of gestation, which affects 1-3% of couples (1). In women with a history of recurrent miscarriage, the risk of miscarriage in a subsequent pregnancy is about 40-50%. It has a major influence on the wellbeing and psychosocial status of patients, therefore improved diagnosis and development of treatment strategies is essential (2, 3). 

The risk of miscarriage is enhanced by variety of genetics and environmental factors. Genetic disorders, reproductive tract anatomical pathologies, infectious diseases, endocrine dysfunctions, autoimmune diseases, and thrombophilia are known to be the most important risk factors for RPL (4, 5). Thrombophilia or hypercoagulability is the propensity to develop thrombosis due to an abnormality in the coagulation system. Possible causes of thrombophilia can be either acquired or congenital (6).

Mutation in factor V, methylene tetra hydro folat reductase (MTHFR), the prothrombin or factor II genes, and protein C and S deficiency are the most frequent causes of congenital thrombophilia (7). Several observations have supported the correlation between congenital thrombophilia and recurrent fetal loss. Placental thrombosis and abruption has been found in women with recurrent miscarriage, which suffer from thrombophilia (8, 9). In addition, proinflammatory changes, altered Th1 to Th2 cytokine ratio and complement activation, have been repeatedly found in these women (3) .Normal pregnancies lead to haemostatic changes towards a procoagulatory state. This is followed by an increase concentration of clotting factors and fibrinogen and decrease level of anticoagulant factors with reduced fibrinolytic activity (10). It seems that some of RPL patients are in a permanent acquired procoagulatory state, which fibrin deposits are found in their intervillous space of the placentas (11).

The treatment mainly is aspirin and heparin. Aspirin is an anticoagulant that prevents thrombosis by the increase prostaglandin E_2_. It accelerates blood to placenta, which should be started from the beginning of pregnancy. Heparin has both anticoagulative and anti-inflammatory effects. Heparin does not penetrate the placenta and is harmless for fetus. It should be started at 6^th^ week of gestation after confirmation of a viable pregnancy. Several studies have examined the use of these throughout pregnancy. They have demonstrated improved fetal outcomes, and also prevention of venous thromboembolism in mother during childbirth (12, 13). However, it was not confirmed by all previous study (14). 

This study evaluated the effect of various anticoagulant treatments on the live-birth rate in women with a history of at least two continuous unexplained miscarriages or thrombophilia. It tries to compare two methods of treatment, with aspirin and aspirin plus heparin.

## Materials and methods

This quasi experimental study evaluated 520 women with recurrent pregnancy loss for four years, 2008-2012. They had referred to recurrent abortion clinic of Yazd Reproductive Sciences Institute. Two hundred and fifty two women with unexplained recurrent pregnancy losses included in this study. Inclusion criteria was unexplained recurrent pregnancy loss or women with thrombophilia and exclusion criteria were abnormal karyotypes of each partner, uterine and/or cervical anatomical disorders on pelvic ultrasonography or hysteroscopy, abnormal ovaries function, abnormal endocrine tests, and antiphospholipid syndrome. In the first visit after 2 or more abortion women were evaluated for mentioned problems. When these problems rolled out, treatment started for the women who accept to receive medication (aspirin or aspirin plus heparin). 

Two hundred fifty two women out of 520 women were chosen, and they accepted to be part of this study by sign inform consent. These women divided to two groups. First group, 134 women, received aspirin (80mg daily) for at least two month before pregnancy and after confirmation of a viable pregnancy until 36 weeks of gestation. They were under 25 years old and had two abortions. Second group, 118 women, received aspirin the same as first group plus heparin (5000 unit twice a day) subcutaneously after confirmation of viable pregnancy until 4 weeks after delivery. They had more than two abortions and/or more than 25 years old. 

Patients were explained about these medication complications and treatment was continued by their obstetrics in their pregnancy care unit. Their PTT was checked every month with heparin injections. Primary outcome was live-birth rate or pregnancy past 20 weeks. Secondary outcome was abortion. The Ethic Committee of Yazd Reproductive Sciences Institute confirmed this study. All women were followed during pregnancy to the end or were contacted by telephone every 3 months till the end of pregnancy. 

**Statistical analysis**

The results analyzed by SPSS (version 15) and they were tested by chi-square test. The results were significant in p>0.05.

## Results

The results showed that, 114 women from 134 get pregnant and completed their treatment with aspirin, and 102 women of 118 with aspirin plus heparin. From 36 out of 252, 25 women never used aspirin and/or heparin, which the pregnancy rate in this group was 60%. Eleven women with treatment never get pregnant even with assisted reproductive techniques. In the first group treated with aspirin, 89 had normal delivery or passed 20 weeks of gestation, 2 preterm labors with normal child and 23 miscarriages. 

In second group treated with aspirin plus heparin, 73 had normal delivery or passed 20 weeks of gestation, 5 preterm labors with normal child and 24 miscarriages. From 25 women who get pregnant without any treatment, 10 had normal delivery. Pregnancy rate after treatment with aspirin compare with aspirin plus heparin was not significantly different (chi-square test, p>0.05). 

**Figure 1:**
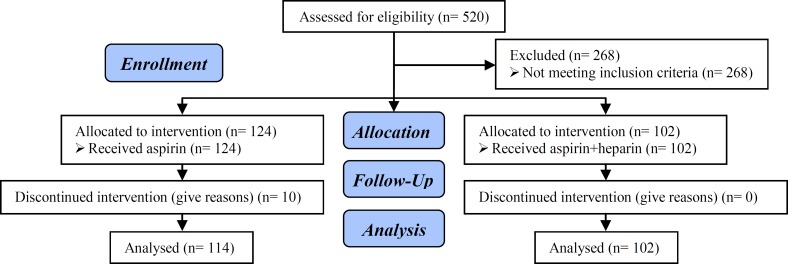
Consort flow diagram

Of 252 women referred to recurrent abortion clinic, 241 (95.6%) became pregnant, and 78.2% of those who became pregnant had a live birth. Live-birth rates were 79.8% with aspirin and 76.4% with aspirin plus heparin. The difference was not significant (p>0.05), but the success rate with aspirin was slightly higher than aspirin plus heparin. (Figure 1)

**Table I:** Successful pregnancy and abortion rate in RPL women after treatment with various anticoagulant agents

**Treatment **	**Normal delivery or passed 20 weeks **	**Preterm labor**	**Abortion **	**Sum **	**Percentage of success**
Aspirin (N)	89	2	23	114	79.8%
Aspirin + Heparin (N)	73	5	24	102	76.4%
No medications (N)	10	5	10	25	60%

X^2^ test.

## Discussion

Present results showed that pregnancy rate with anticoagulant therapy could treat women with unexplained recurrent pregnancy loss, which results of using aspirin or heparin were nearly the same? The hypothesis that women with unexplained recurrent pregnancy loss might benefit from aspirin, heparin, or both was based on a presumption that this condition might be caused by thrombosis in decidual vessels (15, 16). However, antithrombotic therapy with heparin do not recommended for unexplained RPL in general. It is suggested for those with heritable thrombophilia, or with three or more losses, or second trimester losses that might benefit (17, 18). 

Laude *et al* found that levels of circulating procoagulant microparticles were higher in women with recurrent pregnancy loss than in control subjects (19). In Dolitzky *et al* study, 104 patients were randomized, 54 treated with enoxaparin and 50 with aspirin. Ninety four percent had successful pregnancy with enoxaparin and 81% with aspirin (12). They showed higher rate of success than present study, which could cause from patient selection. Also in a study aspirin or aspirin plus low molecular weight heparin were prescribed for 364 women with unexplained recurrent miscarriage, and live birth rates were not different significantly among two groups (20). In Tzafettas *et al* study success rate in two groups treated by aspirin or aspirin plus heparin were equal and was 92% (21). 

Degiannidis *et al* found that low-molecular-weight heparin and low-dose aspirin daily during pregnancy appear to have a favorable effect on pregnancy outcome in selected women with RSAs and acquired or inherited thrombophilia (22). Discussion on the efficacy of aspirin and heparin progressed with recently published randomized-controlled trials (23). However, some study suggested anticoagulant therapy is not effective in idiopathic recurrent abortion treatment (13, 24).

## Conclusion

In conclusion, present study did not find significant difference between the effectiveness of aspirin compare with aspirin and heparin in treatment of idiopathic recurrent abortion with unknown reason. As the success percentage of aspirin is higher and due to its consumption that is once a day orally and easily, it is more preferred to use only aspirin in comparison with the hypodermic treatment of heparin which is done twice a day. In the future, this is recommended to use aspirin and/or heparin according to the recurrent pregnancy loss characteristic, like number of abortion, age and so on. 
